# Prevalence and Periodontal Conditions of Developmental Grooves in an Italian School of Dentistry and Dental Hygiene: A Cross-Sectional Study

**DOI:** 10.3390/ijerph19074047

**Published:** 2022-03-29

**Authors:** Giovanna Laura Di Domenico, Simone Fabrizi, Paolo Capparè, Maria Teresa Sberna, Massimo de Sanctis

**Affiliations:** 1 Department of Dentistry, IRCCS San Raffaele Hospital, 20132 Milan, Italy; giovannalauradd@gmail.com (G.L.D.D.); simonefabrizi@gmail.com (S.F.); sberna.mariateresa@hsr.it (M.T.S.); desanctis.massimo@hsr.it (M.d.S.); 2 Dental School, IRCCS San Raffaele Hospital, Vita-Salute University, 20132 Milan, Italy

**Keywords:** radicular grooves, prevalence, risk indicators

## Abstract

Background: The aim of this cross-sectional study was to (i) determine the prevalence and distribution of developmental grooves in a young population and (ii) to evaluate the local periodontal conditions. Methods: Two hundred and fifty-one students with a mean age of 22.9 ± 4.7, attending the School of Dentistry and Dental Hygiene of Vita-Salute San Raffaele University (Milan, Italy) were included. The subjects underwent a clinical evaluation by two calibrated examiners. The following clinical parameters were recorded for each site presenting a radicular groove and for each corresponding site on an adjacent tooth used as control: probing pocket depth, plaque index, bleeding on probing, recession depth. Results: The prevalence of radicular grooves at patient and tooth level was 15.9% and 5%, respectively. When compared to control sites, the number of teeth with a radicular groove that presented plaque and bleeding on probing was higher. The logistic regression analysis showed that the presence of radicular grooves was significantly associated with the presence of plaque (OR, 6.14, *p* < 0.001) and of bleeding on probing (OR, 2.91, *p* = 0.01). Conclusions: The presence of radicular grooves increases the possibility of developing gingival inflammation by acting as a plaque retentive factor.

## 1. Introduction

The oral microbiota is the only accepted etiological factor associated with periodontal diseases. Loe and co-workers demonstrated, in an experimental gingivitis study, a direct relationship between bacterial plaque accumulation and the onset of gingivitis [1]. If left untreated, gingivitis may, in susceptible subjects and under unfavorable periodontal conditions, progress into periodontitis [2,3].

Gingival health can be impaired by local risk factors, known as predisposing factors, such as dental plaque retention factors, which facilitate plaque accumulation at the gingival margin, enabling biofilm adherence and maturation and increasing the difficulty of mechanical plaque removal [4].

Among these factors, physiological variation and anomalies of morphology/anatomy of teeth have been described as local conditions that promote the onset of periodontal diseases. The first ones include the variations in the anatomical characteristics of radicular surfaces, such as the length, the width, the degree of root taper, the presence of root concavity, the root proximity, whereas the second ones, include marginal ridge discrepancies, cervical enamel projections, pits and grooves on tooth surfaces [5,6,7,8,9].

The radicular groove (RG) is a developmental anomaly in which an infolding of the inner enamel epithelium and Hertwig’s epithelial root sheath create a groove that passes from the cingulum of maxillary incisors apically onto the root. These grooves can be found on the buccal or lingual/palatal side [10].

The prevalence of radicular grooves described in previous studies ranges from approximately 2 to 10% for palatal and from 4 to 5% for buccal grooves. Differences in prevalence among studies could be attributed to disparate study methodologies and/or found in, genetic diversities between the examined populations [10,11,12,13,14,15,16].

Radicular grooves make dental plaque removal difficult for the patient and favor bacterial accumulation. Moreover, they have been associated with an increased plaque index (PI), bleeding index (BI), and probing pocket depth (PPD) [10,11,12,13,14,15,16].

Recent studies show that the treatment of palatal or vestibular/gingival grooves often requires a multidisciplinary approach in order to restore optimal periodontal, mucogingival and aesthetic conditions [17,18,19,20,21,22]. However, these treatments may sometimes result in being unpredictable and therefore require particular technical skills [23,24].

In any case, an early diagnosis of palatal or buccal/gingival grooves can reduce the amount of treatment need for the resolution of periodontal and dental lesions. Therefore, it is important to identify and correct, at an early stage, periodontal and dental alterations resulting from the presence of developmental grooves, especially in young subjects.

The present study attempts to evaluate the prevalence and distribution of developmental grooves in a sample of students of Dentistry and Dental Hygiene of the Vita-Salute San Raffaele University (Milan, Italy) and to observe their impact on periodontal health.

## 2. Materials and Methods

The present study was a mono-centric cross-sectional investigation, performed in accordance with the Helsinki Declaration of Human Studies. All procedures were submitted and approved by the Ethics Committee of Vita-Salute San Raffaele University (on 18 May 2017 with protocol number “REC 1 Protocol V2”).

For sample size calculation, the mean prevalence of radicular grooves (5%) reported by previous studies was used as reference. The sample size was calculated to estimate the population prevalence of radicular grooves with 5% precision and 95% confidence level. A minimum sample size of 234 subjects was required to achieve the necessary confidence level.

All students, who had attended Dentistry and Dental Hygiene courses in the Vita-Salute San Raffaele University (Milan, Italy) in the period between September and December 2017, were considered for the inclusion. Each subject was invited to participate in the investigation and was informed about all its pertinent aspects.

The clinical examination was performed by two calibrated investigators (S.F. and P.C.) using a periodontal probe, in order to assess the presence of radicular grooves. For each subject, full mouth plaque score (FMPS) and full mouth bleeding score (FMBS) were reported. The following clinical parameters were recorded for each site presenting a radicular groove and for each corresponding site on an adjacent tooth used as control:

- Probing pocket depth (PPD), value 0 was assigned when the probe revealed a sulcus ≤2 mm, value 1 when the probe reported a sulcus > 2 mm;
- Bleeding on probing (BoP), measured as presence/absence;
- Presence of plaque (PI), measured as presence/absence;
- Recession depth (REC), reported in mm.

Descriptive statistics are presented as frequencies, means and standard deviations. Prevalence of radicular grooves was calculated at patient and tooth level. Extent of the defects was assessed as the proportion of affected tooth in patients with the condition.

Fisher’s exact test was used to examine the differences of categorical variables between teeth with or without grooves, while Student *t*-test was used for continuous variables in order to compare the means of the two groups. A linear regression analysis was conducted to determine if the presence of radicular grooves represented a risk indicator for the examined periodontal parameters and if sociodemographic data of the patients were associated with the presence of this tooth anomaly. The statistical analyses were performed using the software package (SPSS, Statistical Package for the Social Sciences; SPSS Inc., Chicago, IL, USA).

## 3. Results

A total of 251 students participated in the examination. Ten students of the dental school and three students of the school of dental hygiene refused to participate in the study. The sample included 96 males (38%) and 155 females (62%). The main part of 69.7% (*n* = 175) of our sample consisted of students of the dental school, whereas 30.3% (*n* = 76) attended the school of dental hygiene. The mean age of the sample of patients was 22.9 ± 4.7 years (range, 19–50; median, 22). The mean % of FMPS and FMBS was 31.1 ± 19.1 and 21.8 ± 19.6, respectively.

### 3.1. Prevalence and Distribution

Forty out of 251 subjects (15.9%) showed at least one radicular groove on the surface of a maxillary incisor.

Fifty radicular grooves were identified on 1008 maxillary incisors (5%), of these, 46% (*n* = 23) were found on the buccal surface and 54% (*n* = 27) on the palatal surface (Table 1), with a distribution of 4.4% (*n* = 22) in central incisors and of 5.5% (*n* = 28) in lateral incisors.

### 3.2. Periodontal Conditions

Overall, a total of 100 teeth were examined. Out of the 32 teeth with the presence of plaque and 47 with BoP, 25 (78%) and 30 (64%) presented a radicular groove, respectively (Table 2).

Fisher’s exact test was used to compare the presence of plaque and of bleeding on probing between teeth presenting radicular grooves and adjacent teeth without grooves used as controls. Both the variables were significantly different between the two groups (*p* < 0.001 and *p* = 0.016, respectively).

The mean PPD was significantly different (*p* < 0.001) between teeth with (1.88 ± 0.82 mm) and without (1.26 ± 0.49 mm) radicular grooves. Furthermore, an increased PPD (>2 mm) was found in 10% of teeth with radicular grooves (Table 2). No teeth presented gingival recessions.

The logistic regression analysis showed that the presence of radicular grooves was significantly associated with the presence of plaque and of bleeding on probing (Table 3). In detail, teeth with at least one radicular groove presented a significantly higher OR for the presence of plaque (OR, 6.14, *p* < 0.001) and for the presence of bleeding on probing (OR, 2.91, *p* = 0.01), but not for PPD > 2 mm (OR, 0.08, *p* = 0.786).

At patient level, neither age nor sex was associated with the presence of radicular grooves (*p* = 0.854 and *p* = 567).

## 4. Discussion

The present study investigated the prevalence of radicular grooves in a sample of Dentistry and Dental Hygiene students and the impact of these developmental anomalies on periodontal health.

At patient level, the prevalence of radicular grooves was 15.9%. This finding was greater than that described by some previous reports. For example, Withers et al. (1981) reported an 8.5% prevalence of palato-gingival grooves in a sample of 531 examined subjects in Texas, while Pecora and da Cruz Filho (1992) found radicular grooves in 3.9% of the 642 Brazilian patients analyzed in their study, especially on the lingual surface of the maxillary lateral incisors (3%). Moreover, Al-Rasheed (2011) reported a prevalence of 10.3% in a sample of 273 Saudi adult male patient. This variability could be attributed to difference in race and genetics. [12,25,26].

Regarding the prevalence at tooth level, the results of the present study are in agreement with those of Kogon et al. (1986), who examined 3168 extracted maxillary incisors, reporting that 4.6% of these teeth presented a palato-radicular groove. On the contrary, our result was greater than that found by Everett and Kramer (1972), who reported a prevalence of radicular grooves of 1.9% [10,11]. The difference may be due to the fact that the authors in their study examined only extracted maxillary lateral incisors and recorded only those grooves involving the palatal radicular surface. Moreover, another reason for the difference may be due to the fact that the present study evaluated grooves present both on buccal and palatal surfaces.

Nevertheless, other researchers have reported higher occurrence rates. Storrer et al. (2006) reported a prevalence rate of 9.58% in a survey of 73 extracted maxillary lateral incisors. In a clinical examination of 200 patients, Iqbal et al. (2011) reported a prevalence rate of 10%. Hou et al. (1993) reported a prevalence rate of 18.1% in clinical examinations of 404 maxillary incisors in 101 individuals. This variance in prevalence rates could be due to different diagnostic criteria or examination methodologies (e.g., survey of extracted teeth vs. clinical examination) or to ethnic/racial differences, which would suggest a genetic relationship [13,27,28].

The results of the present study revealed a greater prevalence of RG in the palatal region of affected teeth (n = 27, 54%) than in the vestibular region (*n* = 23, 46%), and more radicular grooves were found in the palatal area of lateral incisors (*n* = 23) than of central incisors (*n* = 4). These findings are in agreement with Kogon (1986), who reported 100 radicular grooves (5.6%) on lateral incisors and 47 (3.4%) on central ones, but are in disagreement with the results of Lee et al. (1968) [11,29].

The question of whether the presence of radicular grooves influenced local periodontal conditions was addressed in the current study.

The data showed that teeth with a radicular groove were six times more likely to accumulate plaque (OR, 6.14, *p* < 0.001) and three times more likely to develop inflammation (BoP) (OR, 2.91, *p* = 0.01). These findings agree with Withers et al. (1981) and Hou et al. (1993). Grooves may facilitate plaque growth by providing surface areas sheltered from the efforts of maintenance of oral hygiene. This may explain the negative effects of grooves and their tendency to cause clinical attachment loss, as reported in the 2017 World Workshop on the Classification of Periodontal and Peri-Implant Diseases and Conditions [12,28,29,30,31].

The findings obtained from the present study indicate that gingival sulcus at the sites with grooves demonstrated significantly higher probing depth compared to teeth without grooves. This result is in accordance with the findings of Withers et al. (1981), Hou et al. (1993), and Al-Rasheed (2011), that reported a statistical difference in the mean probing depth between teeth with radicular grooves and those with no grooves [12,26,28].

In any case, the respective mean depth of the two groups was still in the physiological range: in fact, an increased PPD (>2 mm) was found in only 10% of teeth with anomalies. However, it must be taken into account that the examined population was of students with a mean age of 22.9 ± 4.7 years and that a periodontal lesion had had time to develop.

The limits of the present study are inherent to the cross-sectional design. Indeed, no data on the incidence and the progression of these anomalies may be anticipated since no longitudinal evaluation was performed. Monitoring the periodontal condition of teeth affected by radicular grooves would greatly help in the planning of an appropriate clinical treatment approach.

Another limitation of the current investigation is inherent to the study population. Indeed, this specific population has an age that varies between 19 and 50 (median: 22) and presents specific concerns on oral health, as compared to a normal sample of patients.

Nevertheless, the present study showed that radicular grooves are important factors that have an impact on periodontal conditions, since they favor plaque accumulation and inflammation. This is an important observation in terms on clinical relevance and it may indicate that, independently of the young age of patients, this tooth anomaly is an important factor that may have an impact on the occurrence of gingival inflammation and hence the need to be controlled.

## 5. Conclusions

In summary, data obtained from the present study show a prevalence of 15.9% of radicular grooves in a young population of dentistry and oral hygiene Italian students. Moreover, a clear association between the presence of radicular grooves and the presence of plaque and bleeding on probing was found, demonstrating that radicular grooves can increase the possibility of developing gingival inflammation by acting as a plaque retentive factor.

## Figures and Tables

**Table 1:** Distribution of radicular grooves.

	Buccal Side	Palatal Side	Total
Central incisor	18	4	22
Lateral incisor	5	23	28
Total	23	27	50

**Table 2:** Plaque Index, Bleeding on Probing (BoP) and Probing Pocket Depth (PPD) in teeth with and without radicular grooves.

	With Grooves (*n* = 50)	Without Grooves (*n* = 50)	Total
Presence of plaque	25	7	32
Absence of plaque	25	43	68
Presence of BoP	30	17	47
Absence of BoP	20	33	53
PPD ≤ 2 mm	45	50	95
PPD > 2 mm	5	0	5
Mean (mm)	1.88 ± 0.82	1.26 ± 0.49	/
Presence of gingival recession	0	0	0
Absence of gingival recession	50	50	100

**Table 3:** Logistic regression with presence of radicular groove as dependent variable.

Outcome variable: Presence of plaque	OR	95% CI	*p*-value
Presence of radicular groove	6.14	2.32–16.24	<0.001 *
Outcome variable: Presence of BoP	OR	95% CI	*p*-value
Presence of radicular groove	2.91	1.29–6.57	0.01 *
Outcome variable: Presence of PPD > 2 mm	OR	95% CI	*p*-value
Presence of radicular groove	0.08	0.004–1.52	0.786

OR: Odds ratio; BoP: Bleeding on Probing; * = statistically significant.

## Data Availability

The data presented in this study are available on request from the corresponding author.

## References

[1] Löe H., Theilade E., Jensen S.B. (1965). Experimental Gingivitis in Man. J. Periodontol..

[2] Loe H., Anerud A., Boysen H., Morrison E.C. (1986). Natural history of periodontal disease in man. Rapid, moderate and no loss of attachment in Sri Lankan laborers 14 to 46 years of age. J. Clin. Periodontol..

[3] Neely A.L., Holford T.R., Löe H., Anerud A., Boysen H. (2001). The Natural History of Periodontal Disease in Man. Risk Factors for Progression of Attachment Loss in Individuals Receiving No Oral Health Care. J. Periodontol..

[4] Chapple I.L.C., Mealey B.L., Van Dyke T.E., Bartold P.M., Dommisch H., Eickholz P., Geisinger M.L., Genco R.J., Glogauer M., Goldstein M. (2018). Periodontal health and gingival diseases and conditions on an intact and a reduced periodontium: Consensus report of workgroup 1 of the 2017 World Workshop on the Classification of Periodontal and Peri-Implant Diseases and Conditions. J. Clin. Periodontol..

[5] Di Angelo L., Di Stefano P., Bernardi S., Continenza M. (2016). A new computational method for automatic dental measurement: The case of maxillary central incisor. Comput. Biol. Med..

[6] Aykol-Sahin G., Arsan B., Altan-Koran S.M., Huck O., Baser U. (2021). Association between root taper and root proximity of single-rooted teeth with periodontitis: A cone-beam computed tomographybased study. Odontology.

[7] Bačić M., Karakaš Z., Kaić Z., Šutalo J. (1990). The Association Between Palatal Grooves in Upper Incisors and Periodontal Complications. J. Periodontol..

[8] Leknes K.N., Lie T., Selvig K.A. (1994). Root Grooves: A Risk Factor in Periodontal Attachment Loss. J. Periodontol..

[9] Shpack N., Dayan T., Mass E., Vardimon A.D. (2007). Labial cervical vertical groove (LCVG) distribution and morphometric characteristics. Arch. Oral Biol..

[10] Everett F.G., Kramer G.M. (1972). The Disto-lingual Groove in the Maxillary Lateral Incisor; A Periodontal Hazard. J. Periodontol..

[11] Kogon S.L. (1986). The Prevalence, Location and Conformation of Palato-Radicular Grooves in Maxillary Incisors. J. Periodontol..

[12] Withers J.A., Brunsvold M.A., Killoy W.J., Rahe A.J. (1981). The Relationship of Palato-Gingival Grooves to Localized Periodontal Disease. J. Periodontol..

[13] Storrer C.M., Sanchez P.L., Romito G.A., Pustiglioni F.E. (2006). Morphometric study of length and grooves of maxillary lateral incisor roots. Arch. Oral Biol..

[14] Mass E., Aharoni K., Vardimon A.D. (2005). Labial-cervical-vertical groove in maxillary permanent incisors--prevalence, severity, and affected soft tissue. Quintessence Int..

[15] Gound T.G., Maze G.I. (1998). Treatment options for the radicular lingual groove: A review and discussion. Pract. Periodontics Aesthetic Dent..

[16] Tan X., Zhang L., Zhou W., Li Y., Ning J., Chen X., Song D., Zhou X., Huang D. (2017). Palatal Radicular Groove Morphology of the Maxillary Incisors: A Case Series Report. J. Endod..

[17] Cho Y.-D., Lee J.-E., Chung Y., Lee W.-C., Seol Y.-J., Lee Y.-M., Rhyu I.-C., Ku Y. (2016). Collaborative Management of Combined Periodontal-endodontic Lesions with a Palatogingival Groove: A Case Series. J. Endod..

[18] Sooratgar A., Tabrizizade M., Nourelahi M., Asadi Y., Sooratgar H. (2016). Management of an Endodontic-Periodontal Lesion in a Maxillary Lateral Incisor with Palatal Radicular Groove: A Case Report. Iran Endod. J..

[19] Garrido I., Abella F., Ordinola-Zapata R., Duran-Sindreu F., Roig M. (2015). Combined Endodontic Therapy and Intentional Replantation for the Treatment of Palatogingival Groove. J. Endod..

[20] Tabari Z.A., Homayouni H., Pourseyediyan T., Arvin A., Eiland D., Majd N.M. (2016). Treatment of a Developmental Groove and Supernumerary Root Using Guided Tissue Regeneration Technique. Case Rep. Dent..

[21] Sharma S., Deepak P., Vivek S., Dutta S.R. (2015). Palatogingival Groove: Recognizing and Managing the Hidden Tract in a Maxillary Incisor: A Case Report. J. Int. Oral Health.

[22] Castelo-Baz P., Ramos-Barbosa I., Martín-Biedma B., Dablanca-Blanco A.B., Varela-Patiño P., Blanco-Carrión J. (2015). Combined Endodontic-Periodontal Treatment of a Palatogingival Groove. J. Endod..

[23] Forero-López J., Gamboa-Martínez L., Pico-Porras L., Niño-Barrera J.L. (2015). Surgical management with intentional replantation on a tooth with palato-radicular groove. Restor. Dent. Endod..

[24] Zucchelli G., Mele M., Checchi L. (2006). The Papilla Amplification Flap for the Treatment of a Localized Periodontal Defect Associated With a Palatal Groove. J. Periodontol..

[25] Pécora J.D., Filho A.M.C. (1992). Study of the incidence of radicular grooves in maxillary incisors. Braz. Dent. J..

[26] Al-Rasheed A. (2011). Relationship between palate-radicular groove and periodontal health in maxillary lateral incisors. Pak. Oral Dent. J..

[27] Iqbal N., Tirmazi S.M., Majeed H.A., Munir M.B. (2011). Prevalence of palate-gingival groove in maxillary lateral incisors. Pak. Oral Dent. J..

[28] Hou G.L., Tsai C.C. (1993). Relationship between palato-radicular grooves and localized periodontitis. J. Clin. Periodontol..

[29] Lee K.W., Lee E.C., Poon K.Y. (1968). Palato-gingival grooves in maxillary incisors. A possible predisposing factor to localised periodontal disease. Br. Dent. J..

[30] Jepsen S., Caton J.G., Albandar J.M., Bissada N.F., Bouchard P., Cortellini P., Demirel K., de Sanctis M., Ercoli C., Fan J. (2018). Periodontal manifestations of systemic diseases and developmental and acquired conditions: Consensus report of workgroup 3 of the 2017 World Workshop on the Classification of Periodontal and Peri-Implant Diseases and Conditions. J. Clin. Periodontol..

[31] Ercoli C., Caton J.G. (2018). Dental prostheses and tooth-related factors. J. Periodontol..

